# COVID-19 Antibodies in Vaccinated Healthcare Workers: The Security Currency

**DOI:** 10.7759/cureus.23383

**Published:** 2022-03-22

**Authors:** Ali Ammar, Jehangir A Shah, Waqar Khan, Rajesh Kumar, Abdul H Shaikh, Farheen Ali, Mehwish Zehra, Jawaid A Sial, Tahir Saghir, Zahid U Rehman

**Affiliations:** 1 Adult Cardiology, National Institute of Cardiovascular Diseases (NICVD), Karachi, PAK; 2 Cardiology, National Institute of Cardiovascular Diseases (NICVD), Karachi, PAK; 3 Infectious Disease, National Institute of Cardiovascular Diseases (NICVD), Karachi, PAK; 4 Medicine, Jinnah Postgraduate Medical Center, Karachi, PAK; 5 Clinical Research, National Institute of Cardiovascular Diseases (NICVD), Karachi, PAK

**Keywords:** pakistan, antibodies, vaccination, healthcare workers, covid-19

## Abstract

Background

The coronavirus disease 2019 (COVID-19) vaccinations have brought new hope to the world and have a significant psychosocial impact on communities as well as healthcare systems around the globe. This study aimed to assess the antibody titer level among healthcare workers after at least six weeks of the second dose of the COVID-19 vaccine.

Methods

Participants of the study were healthcare workers of a tertiary care cardiac center including doctors, nursing staff, paramedics, and office staff. All participants were fully vaccinated with recommended double dose of available vaccine at least six weeks before the study. A blood sample of five milliliters was collected from all the participants by a trained phlebotomist at a local laboratory, and COVID-19 antibodies titer level was assessed using Food and Drug Administration (FDA) approved kit with a standard range of 1.0. This qualitative assay detects IgG and IgM as total antibodies targeted against nucleocapsid antigen performed on a fully automated cobas® 6000 analyzer (F. Hoffmann-La Roche Ltd, Basel, Switzerland) using electrochemiluminescence technology. COVID-19 antibodies titer levels were categorized as ≤100, 101-250, and >250.

Results

A total of 151 healthcare workers were included, of which 70.2% (106) were male. The history of COVID-19 infection before vaccination was found in 41.1% (62). The mean duration since the last dose of the vaccine was 89.6±40.07 days. In total 71.5% (108) had antibodies titer level of >250, which were mostly found in participants of younger age and who had previous COVID-19 infection. However, antibodies titer level of >250 were observed in 84% (21/25) at 61 to 90 days of vaccination, which declined to 80% (20/25) after 91 to 120 days and to 57.1% (32/56) after >120 days of vaccination.

Conclusions

Good antibodies titer levels were observed in vaccinated healthcare workers, especially in those who were younger and had previous COVID-19 infection.

## Introduction

According to the World Health Organization (WHO), coronavirus disease 2019 (COVID-19) infected more than 464.5 million individuals globally as of March 17, 2022, and has claimed around 6 million lives since the very first case reported in December 2019 [1]. COVID-19 caused an unprecedented scenario with significant health, security, and financial implications, especially in developing countries [2,3]. The frontline healthcare workers remain the most vulnerable and affected segment throughout this pandemic. Hence, the arrival of long-awaited efficacious vaccines was well welcomed. Healthcare workers were the first to get vaccinated in most healthcare systems across the globe [4].

According to the WHO COVID-19 dashboard, as of March 17, 2022, a total of 10,925 million doses of vaccine have been administered [1]. The vaccination strategy for most of the currently approved vaccines is a two-stage "prime and boost" strategy with the first dose followed by the second at least three weeks later [5,6]. Clinical data from the initial experience of vaccination reported good effectiveness in preventing symptoms [7].

The COVID-19 vaccinations have brought new hope to the world and have a significant psychosocial impact on communities and healthcare systems around the globe. However, data regarding the clinical effectiveness of the COVID-19 vaccine for improving immunity levels in our population is very limited. Therefore, this study was designed to assess the antibody titer level among frontline healthcare workers after at least six weeks of the second dose of the COVID-19 vaccine.

## Materials and methods

This cross-sectional study was conducted at a tertiary care cardiac center of Karachi, Pakistan, between July 1, 2021, and August 15, 2021. The study was approved by the ethical board of the National Institute of Cardiovascular Diseases, Karachi, Pakistan (ERC-65/2021). Participants of the study were healthcare workers working in a public section tertiary care cardiac center of Karachi, Pakistan, in various designations, including doctors, nursing staff, paramedics, and office staff. Study inclusion criteria were healthcare workers of either gender above 18 years of age, fully vaccinated with recommended double dose of available vaccine at least six weeks before the study. Healthcare workers who did not give consent to assess COVID-19 antibody titers or participate in the study were excluded. Study participants were assessed using the non-probability convenient sampling technique.

The purpose and process of the study were explained to all the participants and demographic data such as gender and age were obtained. Data regarding pre-existing co-morbidities were also obtained, which include the history of chronic obstructive pulmonary disease (COPD)/asthma, diabetes, hypertension, obesity, and smoking status. Vaccination information was obtained and verified from government-approved vaccination certificates. Vaccination-related data included type of vaccine (Sinopharm, China National Pharmaceutical Group Corporation, Beijing, China; Sinovac, Sinovac Biotech Ltd, Beijing, China; CanSino-Bio Convidecia™; CanSino Biologics Inc., Tianjin, China), duration since final dose (in days), pre-and post-vaccination COVID-19 infection status, and severity. Pre- and post-vaccination COVID-19 infection status was verified through a polymerase chain reaction (PCR) test record.

A blood sample of five milliliters was collected from all the participants by a trained phlebotomist at a local laboratory, and COVID-19 antibodies titer level was assessed using Food and Drug Administration (FDA) approved kit with a standard range of 1.0. This qualitative assay detects IgG and IgM as total antibodies targeted against nucleocapsid antigen performed on a fully automated cobas® 6000 analyzer (F. Hoffmann-La Roche Ltd, Basel, Switzerland) using electrochemiluminescence technology.

Antibodies titer levels were categorized into three groups, less than or equal to 100, between 101 and 250, and more than 250. Collected information was recorded using a predefined proforma designed using Google Forms (Google LLC, Menlo Park, California, United States (US)).

IBM SPSS Statistics for Windows, Version 21.0 (Released 2012; IBM Corp., Armonk, New York, US) was used to analyze data. Mean ± SD was computed for the continuous variables such as the age of the participant and duration since final dose of vaccination (days), other categorical variables were expressed as frequency and column percentage (Col. %) or row percentage (Row %). The strength of association between COVID-19 antibodies titer level and baseline characteristics was assessed by conducting an appropriate Chi-square test/analysis of variance (ANOVA). Statistical significance was taken as p-value ≤ 0.05.

## Results

A total of 151 fully vaccinated healthcare workers participated in this study, 70.2% (106) of whom were male, and a majority, 65.6% (99), were under 35 years of age. The history of COVID-19 infection before vaccination was found in 41.1% (62). The mean duration since the last dose of the vaccine was 89.6 ± 40.07 days, and most of the participants, 79.5% (120), were vaccinated with Sinopharm. Vaccine-related complications were reported by eight (5.3%) participants, which included pain at the site of vaccination (four), fever (four), flu (two), and myalgia (five). A majority of the participants, 71.5% (108), had antibodies titer levels of >250, and 11.9% (18) participants were observed to have antibodies titer levels of ≤ 100. Good distribution of antibodies titer level was associated with younger age and previous COVID-19 infection. However, antibodies titer level of >250 was observed in 84% (21/25) at 61 to 90 days of vaccination, which declined to 80% (20/25) after 91 to 120 days and 57.1% (32/56) after >120 days of vaccination (Table 1).

**Table 1:** Distribution of COVID-19 antibodies titer levels by various baseline characteristics * Significant at 5% **Based on patients with COVID-19 infection history COVID-19: coronavirus disease 2019; Col %: column percentage

Characteristics	Total (Col. %)	Antibodies Titer level (Row %)			P-value
		≤ 100	101 to 250	>250	
Total (N)	151	18 (11.9%)	25% (16.6%)	108 (71.5%)	-
Gender					
Male	70.2% (106)	9.4% (10)	17.9% (19)	72.6% (77)	0.315
Female	29.8% (45)	17.8% (8)	13.3% (6)	68.9% (31)	
Age (years)	34.92 ± 7.64	39.72 ± 9.84	34.96 ± 7.25	34.11 ± 7.08	0.015*
≤ 35 years	65.6% (99)	8.1% (8)	16.2% (16)	75.8% (75)	0.034*
36 to 45 years	25.8% (39)	12.8% (5)	17.9% (7)	69.2% (27)	
> 45 years	8.6% (13)	38.5% (5)	15.4% (2)	46.2% (6)	
Type of vaccine					
Sinopharm	79.5% (120)	13.3% (16)	16.7% (20)	70% (84)	0.836
Sinovac	19.9% (30)	6.7% (2)	16.7% (5)	76.7% (23)	
Cansino-Bio	0.7% (1)	0% (0)	0% (0)	100% (1)	
Days since vaccination	89.6 ± 40.07	113.78 ± 38.26	90.76 ± 38.44	85.31 ± 39.62	0.019*
≤ 60 days	29.8% (45)	6.7% (3)	15.6% (7)	77.8% (35)	0.034*
61 to 90 days	16.6% (25)	0% (0)	16% (4)	84% (21)	
91 to 120 days	16.6% (25)	8% (2)	12% (3)	80% (20)	
> 120 days	37.1% (56)	23.2% (13)	19.6% (11)	57.1% (32)	
Previous COVID-19 infection					
No	58.9% (89)	15.7% (14)	24.7% (22)	59.6% (53)	<0.001*
Yes	41.1% (62)	6.5% (4)	4.8% (3)	88.7% (55)	
**Infection severity					
Non-Severe	33.8% (51)	5.9% (3)	3.9% (2)	90.2% (46)	0.897
Severe	6.6% (10)	10% (1)	10% (1)	80% (8)	
Critical	0.7% (1)	0% (0)	0% (0)	100% (1)	
Post vaccination COVID-19 infection					
No	92.7% (140)	12.9% (18)	17.1% (24)	70% (98)	0.296
Yes	7.3% (11)	0% (0)	9.1% (1)	90.9% (10)	

The most common co-morbid condition was obesity observed in 12.6% (19). Eight (5.3%) participants were smokers, eight (5.3%) had COPD/asthma, five (3.3%) were hypertensive, and two (1.3%) were diabetic. No apparent statistically significant association was observed between the distribution of antibodies titer level and co-morbid conditions (Table 2).

**Table 2:** Distribution of COVID-19 antibodies titer levels by co-morbid condition status COPD: chronic obstructive pulmonary disease; COVID-19: coronavirus disease 2019; Col %: column percentage

Characteristics	Total (Col. %)	Antibodies Titer level (Row %)			P-value
		≤ 100	101 to 250	>250	
Total (N)	151	18 (11.9%)	25% (16.6%)	108 (71.5%)	-
Hypertension					
No	96.7% (146)	12.3% (18)	15.8% (23)	71.9% (105)	0.297
Yes	3.3% (5)	0% (0)	40% (2)	60% (3)	
Diabetes Mellitus					
No	98.7% (149)	12.1% (18)	16.1% (24)	71.8% (107)	0.419
Yes	1.3% (2)	0% (0)	50% (1)	50% (1)	
Smoking					
No	94.7% (143)	11.2% (16)	16.8% (24)	72% (103)	0.498
Yes	5.3% (8)	25% (2)	12.5% (1)	62.5% (5)	
Obesity					
No	87.4% (132)	11.4% (15)	16.7% (22)	72% (95)	0.856
Yes	12.6% (19)	15.8% (3)	15.8% (3)	68.4% (13)	
COPD/Asthma					
No	94.7% (143)	11.2% (16)	17.5% (25)	71.3% (102)	0.269
Yes	5.3% (8)	25% (2)	0% (0)	75% (6)	

## Discussion

The development of vaccination has brought new hope for humanity against the extremely infectious COVID-19, and mass vaccination is considered the primary strategy to curtail this pandemic [8]. Several vaccines have been given approval for emergency use by the regulatory authorities. The results from the mass vaccination campaigns are promising with high protection against disease with the recommended two doses of the vaccine. In the current research, we aimed to assess the antibody titer level in healthcare workers who have completed the recommended two doses of vaccine at least six weeks before the study. Vaccine-related complications were reported by 5.3% of the participants. A majority of the vaccinated healthcare workers had suitable antibodies titer levels, and higher antibodies titer levels were associated with younger age and previous COVID-19 infection. Moreover, a decline in antibodies titer level was observed with an increase in duration since the last dosage with a maximum of 84% of the participants with >250 antibodies titer levels at 61 to 90 days and 57.1% after more than 120 days of vaccination. Even though most of the patients developed suitable antibodies, around 7.3% (11) of the participants did develop COVID-19 infection post-vaccination.

Nearly 80% of the participants in our study were vaccinated with Sinopharm. The Sinopharm vaccine has been shown to have neutralizing antibody response for COVID-19 with a low adverse event rate. However, extensive data regarding the safety and efficacy of other available types of the vaccine have been reported, but only a few studies have been conducted for the Sinopharm vaccine [9-11]. Fear of unknown side effects was a significant driver for vaccine hesitancy in a survey conducted in United Arab Emirates (UAE). Similar to ours, the study further reported injection site pain (42.2%), fatigue (12.2%), and headache (9.6%) as common complications after vaccination [12]. Shapiro et al. [13] conducted a study to estimate the efficacy of various vaccines being rolled out globally; these included Sinopharm, Sinovac, Pfizer-BioNTech (Pfizer Inc., New York, US; BioNTech SE, Mainz, Germany), Sputnik (Gamaleya Research Institute, Moscow, Russia), AstraZeneca (AstraZeneca plc, Cambridge, United Kingdom), Moderna (Moderna, Inc., Cambridge, Massachusetts, US), Johnson & Johnson (Johnson & Johnson, New Jersey, US), and Novavax (Novavax, Inc., Maryland, US). It determined an average efficacy of 85% against disease with confirmed infection, 84% against infection, and 54% for transmission to others. Another review reported 100% efficacy of Sinopharm, Sputnik V, and AstraZeneca in prevention against severe COVID-19 [14].

Now that the mass vaccination strategy is implemented; the ultimate goal is to achieve herd immunity; based on Ro value for the infection, nearly 75-90% vaccination coverage is necessary to attain herd immunity against COVID-19 [15]. However, vaccine hesitancy with refusal to get vaccinated due to various myths, rumors, and untrue speculations are deterrents to mass COVID-19 vaccination strategies [16,17]. Along with logistic requirements to ensure mass vaccination, appropriate strategies are also needed to address the misconceptions regarding the development of the vaccine to curtail the transmission of COVID-19.

A very recent study from England on 212,102 vaccinated individuals by Ward et al. reported a peak positive antibody rate at four to five weeks of the first dose [18]. Similar to our observations, antibody positivity was higher in female patients and patients with a history of COVID-19 infection, while antibody positivity decreased with age. Lower antibody positivity was observed in smokers, obese individuals, and recipients of transplants [18]. The study further reported detectable antibodies in a significant number of individuals up until ten weeks after the second dose of vaccine. However, implications of antibody positivity are not fully understood [19]; antibody-positive test does not guarantee protection, just as the absence of antibodies does not, technically, means vulnerability to COVID-19 infection as 11 fully vaccinated subjects with suitable antibodies in our study got infected even after vaccination. However, Earle et al. [20] summarized the evidence for post-immunization antibody titers as a correlate of protection against COVID-19 infection with a correlation coefficient of 0.93 between efficacy and binding antibody titer and 0.79 between efficacy and neutralizing titer.

## Conclusions

Good antibodies titer levels were observed in healthcare workers vaccinated with a recommended dose of vaccine. High antibody titers were associated with younger age and previous COVID-19 infection. A decline in antibodies titer level was observed with an increase in duration since the last dosage with maximum positivity at 61 to 90 days. Hence, further studies are required to assess the need for a booster dose to maintain the immunity level.
